# Direct genotyping of Toxoplasma gondii from amniotic fluids based on B1 gene polymorphism using minisequencing analysis

**DOI:** 10.1186/1471-2334-13-552

**Published:** 2013-11-19

**Authors:** Jean-Marc Costa, Alexandre Alanio, Sandrine Moukoury, Vincent Clairet, Monique Debruyne, Jean-Dominique Poveda, Stéphane Bretagne

**Affiliations:** 1 Laboratoire CERBA, Paris, Cergy-Pontoise, France; 2 Laboratoire de Parasitologie-Mycologie; AP-HP, Groupe Hospitalier Saint-Louis-Lariboisière-Fernand-Widal, Paris, France; 3 Université Paris-Diderot, Sorbonne Cité, Paris, France

**Keywords:** Toxoplasma gondii, P30 gene, B1 gene, AF487550 repeated element, Genotyping, Minisequencing analysis

## Abstract

**Background:**

Because some *Toxoplasma gondii* genotypes may be more virulent in pregnant women, discriminating between them appears valuable. Currently, the main genotyping method is based on single copy microsatellite markers, which limit direct genotyping from amniotic fluids (AFs) to samples with a high parasitic load. We investigated whether the multicopy gene B1 could type the parasite with a higher sensitivity. To estimate the amplifiable DNA present in AFs, we first compared three different PCR assays used for *Toxoplasma* infection diagnosis: the P30-PCR, targeting the single copy gene P30; the B1-PCR, targeting the repeated *B1* gene; and RE-PCR, targeting the repeated element.

**Results:**

Of the 1792 AFs analyzed between 2008 and 2011, 73 were RE-PCR positive. Of those, 49 (67.1%) were P30-PCR and B1-PCR positive, and 14 (19.2%) additional AFs were B1-PCR positive only.

All 63 BI-positive AFs (France n = 49; overseas n = 14) could be genotyped based on an analysis of eight nucleotide polymorphisms (SNPs) located within the *B1* gene. Following high-resolution melting (HRM) analysis, minisequencing was carried out for each of the eight SNPs. DNA from six reference strains was included in the study, and AFs were assigned to one of the three major lineages (Types I, II, and III). In total, 26 genotypes were observed, and the hierarchical clustering distinguished two clades in lineages II (IIa, n = 30 and IIb, n = 4) and III (IIIa n = 23 and IIIb n = 6). There was an overrepresentation of overseas isolates in Clade IIb (4/4, 100%) and Clade IIIa (8/22; 36.4%) (p <0.0001), whereas medical interruption and fetal death were overrepresented in Clade IIb (2/4, 50%) and Clade IIIa (4/23, 17.4%) (p = 0.049).

**Conclusions:**

Although the current genotyping system cannot pretend to replace multilocus typing, we clearly show that targeting the multicopy *B1* gene yields a genotyping capacity of AFs around 20% better than when single copy targets are used. The present genotyping method also allows clear identification of genotypes of potential higher virulence.

## Background

Infection by the protozoan parasite *Toxoplasma gondii* can yield symptoms ranging from asymptomatic infections to miscarriages or meningoencephalitis in immunocompromised patients. In prenatal infections, fetal damages are usually linked to the time of infection during the pregnancy. The earlier on, the more likely miscarriages and severe debilitating infections become [1]. However, genotyping has revealed genetic diversity in this worldwide parasite, and three main lineages (Types I, II, and III) [2], now grouped into 15 haplogroups in six major clades, have been described [3]. Setting aside epidemiological and taxonomic studies, genotype determination is clinically relevant, as some genotypes harbor a specific virulence [4-6]. The need for a simple genotyping system, effective in clinical specimens, is clear.

Microsatellite markers have emerged as a very convenient means for direct genotyping since first reported [7]. Up to 15 markers in a multiplex PCR format are now suggested for genotyping [8]. However, only 62% (15/24) of the AFs sent to the French National Center for toxoplasmosis could be genotyped (http://cnrtoxoplasmose.chu-reims.fr/wp-content/uploads/2012/10/CNR-TOXOPLASMOSE-RAPPORT-ACTIVITES-2011.pdf). The small amount of DNA extracted from clinical samples for single locus typing of microsatellite markers probably explains this dearth of typable isolates. Testing microsatellite markers on samples with low *T. gondii* DNA content can lead to the absence of detectable peaks or peaks of low intensity not easily distinghishable from non specific PCR products [8]. Consequently, if for any reason the non-typable samples correspond to specific genotypes, correlations drawn between symptoms and genotypes or conclusions on epidemiologic trends could be biased.

Several options for improving the rate of typability exist. One possibility is to cultivate the parasite before DNA extraction. However, cultures introduce bias, as not every isolate will grow with the same efficiency. The risk of false positives, especially in the context of a routine laboratory dealing with pregnant women, precludes a nested PCR format [9]. Hence, our choice was to use a multicopy gene as a target. We recently investigated the possibility of genotyping *T. gondii* using the polymorphism of the repeated *B1* gene with high-resolution melting (HRM) analysis and minisequencing (SNaPshot) using reference strains of different types [10]. Our typing system using *B1* was based on a single PCR amplification whose yield is easy to manage with an internal control [11] (as opposed to a multiplex PCR, where failure of one of the primer sets can have multiple causes).

We present here the results of analysis of the amniotic fluids (AFs) sent to our laboratory under suspicion of congenital toxoplasmosis. We added a formal comparison of the three targets used for *Toxoplasma* infection diagnosis: the single copy gene P30 [12], the repeated B1 gene [13], and the repeated element (RE) [14]. Besides this previously unreported comparison, our aim was to find clues indicating the presence of amplifiable DNA in the sample.

## Methods

### Samples

Between 2008 and 2011, 1792 AFs were analyzed for the presence of *T. gondii* DNA. Tests were required because of proven or suspected maternal infection following systematic serological screening for anti-*Toxoplasma* antibodies (n = 1309; 73%), as required by French regulations [15], or for investigation of abnormalities revealed upon ultrasound examination (n = 483; 27%). Samples were sent from 465 different medical centers in France and overseas (French Caribbean, French Guyana, La Réunion Island, New Caledonia, and North Africa). After analysis, the remaining material was stored at -80°C, Informed consent for AF sampling, biochemical and microbiological testing, and data analysis was obtained from all women. In compliance with French regulations of prenatal diagnoses, patients provided informed consent for sample collection and analysis. Local ethics committee approval was not required, as the procedures were part of routine care. The French National Commission on Computing and Freedom was notified of the database (registration number: 1406332).

### Toxoplasma gondii DNA detection

Total DNA was extracted from one ml of each AF using the Total Nucleic Acid Isolation kit (Roche Diagnostics, Meylan, France) on a MagNA Pure Compact apparatus, in accordance with the manufacturer's instructions. DNA was eluted with 100 μl of elution buffer, of which 5 μl were used for each PCR assay. Each AF was tested with three quantitative PCR (qPCR) assays targeting the *P30* gene (P30-PCR; GenBank access number AY187278), the *B1* gene (B1-PCR; GenBank access number AF179871), and the repeated element (RE-PCR; GenBank access number AF487550) [16], as previously described [17].

PCR reactions were carried out in a LightCycler LC480 Instrument (Roche Diagnostics, Meylan, France) in a final volume of 20 μl, using the LC480 Probes Master Kit (Roche Diagnostics, Meylan, France) with 0.5 μM of each primer and 0.25 μM of each probe. Following an initial incubation step of 1 minute at 50°C and a denaturation step of 10 minutes at 95°C, amplification was performed for 50 cycles of denaturation (95°C, 10 seconds, and ramping rate 4.4°C/second), annealing (56°C, 10 seconds, and ramping rate 2.2°C/second), and extension (72°C, 15 seconds, and ramping rate 4.4°C/second).

### Toxoplasma gondii genotyping

If detectable for the *B1* gene, *T. gondii* DNA was genotyped based on an analysis of eight nucleotide polymorphisms (SNPs) located within the *B1* gene, as described elsewhere [10]. All SNPs were studied during a single independent PCR in a final volume of 20 μl with the High Resolution Melting Master Kit (Roche Diagnostics, Meylan, France), 3 mM MgCl_2,_ and each primer (Sigma, Paris, France) at a concentration of 0.5 μM. The reaction mixture was initially incubated for 10-min steps at 95°C. Amplification was performed for 50 cycles of denaturation (95°C for 10 sec; ramp rate, and 4.4°C/s), annealing (60°C for 10 sec; ramp rate, and 2.2°C/s), and extension (72°C for 15 sec; ramp rate, and 4.4°C/s) in a LightCycler 480 Instrument (Roche Diagnostics, Meylan, France). HRM of PCR products was then performed at 95°C for 1 min, at 40°C for 1 min, and with increasing temperature from 65°C to 95°C at a rate of 1°C/s with 25 acquisitions per °C. Following purification of the PCR products with ExoSAP-IT (USB Europe, Staufen, Germany), SNaPshot analysis was carried out using the SNaPshot Multiplex kit (Applied Biosystems, Courtaboeuf, France) for each of the eight examined SNPs, and the reactions were run on an ABI3130XL genetic analyzer and analyzed using the Genescan software. DNA from reference strains for each of the three main lineages, Type I (RH strain), Type II (B7 strain), and Type III (C5 strain), were kindly provided by Asis Khan (David Sibley laboratory), while strains from Africa (Africa 1 [RMS-2003-DJO] and Africa 2 [CCH-2004-NIA]), and the Caribbean (CCH-2005-REN) were purchased from the French National Center of Reference for toxoplasmosis.

### Toxoplasma gondii genotype analysis and genotype-clinical data association

Genotypes were determined for the B1-PCR positive AFs and six reference strains based on determinations of the transition/transversion matrix [18] for each locus tested with SNaPshot analysis. The predominant allele (>50% of the samples) was considered the wild-type allele, and was used as the reference sequence. The polymorphic allele was then classified according to the transition/transversion matrix. In the case of heterozygosity (a double peak at the polymorphic locus), indicating a variable proportion of polymorphic repeated sequence, 50% of the corresponding transition/transversion matrix value was arbitrary attributed.

Based on the combination of the eight markers, we determined the final genotype using an arbitrary implementation. Relatedness between the different genotypes was evaluated based on hierarchical clustering (complete linkage; Euclidian distance metric) using the open-source genomic analysis software MeV v4.6.1 (The TM4 Development Group), obtained from http://mev.tm4.org[19]. The different clades were arbitrarily determined using a threshold of 1.225 (Euclidian).

### Statistical analysis and calculations

The graphs, Chi-squared tests, and ANOVA tests were performed using Prism v4.0 (GraphPAD Software, San Diego, CA). Fold changes from Ct values were calculated following this formula : Fold change (FC) = 2^-ΔCt.^

## Results and discussion

According to the RE-PCR assay, which is used for routine testing in our diagnostic laboratory [16], *T. gondii* DNA was detected in 82/1792 (4.6%) AFs. Of the 82 infected AFs, 73 had enough sample left to be stored at -80°C, and therefore were available for simultaneous comparison of the three DNA targets. The nine AFs with no DNA left for further analysis were from different trimesters (seroconversion in first trimester n = 2, second trimester n = 5, and unknown n = 2) and of different origin (France n = 8; overseas n = 1); therefore, it is improbable that their exclusion impacted the subsequent analysis. The 73 RE-PCR positive AFs came from 65 different cities and 43 different medical centers.

**Figure 1:**
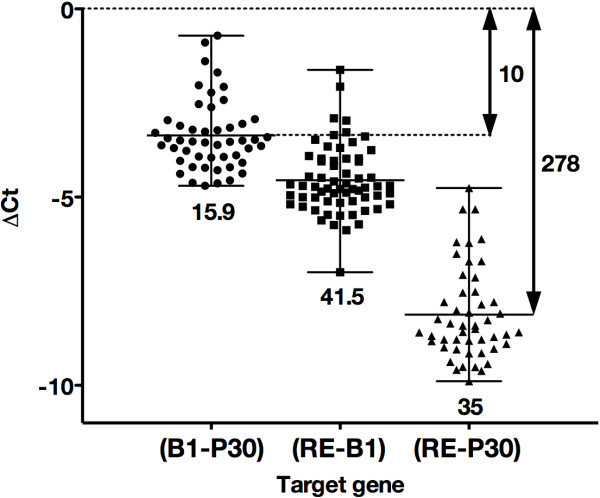
**Dot plot representation of the delta Ct comparing the three PCR assays (P30-PCR, B1-PCR, RE-PCR) for 63 B1-PCR positive AFs.** Horizontal bars represent the mean and range of the delta Ct for all the samples. The repetition of the genes B1 and RE compared to P30, and RE compared to B1, varied among the clinical variation within the fold variation indicated under each dot plot. The mean gain of detection of B1 PCR and RE PCR compared to the P30 PCR are indicated on the right. Note the fourteen AFs that were negative using P30-PCR.

As our main objective was to characterize the infecting *T. gondii* strains directly from the clinical specimen using a multicopy gene target, we performed a comparative study of the three main targets used for detecting *T. gondii* (P30, B1 and RE) to get an idea of the quantity of *T. gondii* DNA in the clinical samples. Indeed, the samples in which no amplification is observed when targeting the single copy gene P30 are expected not to be genotypable with a single copy marker. Although these different targets have already been compared two-by-two [16,20,21], this is the first simultaneous comparison with a consistent number of samples (n = 73). The three qPCR assays were positive in 49/73 (67.1%) samples, the B1-PCR and RE-PCR assays were positive in 14/73 (19.2%) additional samples, and the RE-PCR assays were positive in the remaining 10/73 (13.7%) samples. Figure 1 shows the comparison of quantitative cycle (Cq) function of the target gene for each positive sample (n = 73). The variations of the number of repetitions for the *B1* gene and RE were 15.9 and 35 times, respectively, greater than those of the single copy P30 gene (p <0.0001). Considering variation among the 73 samples, the B1-PCR and RE-PCR increased detection by mean factors of 10 (-3.33 Ct) [range 0.6 to 26] and 278 (-8.12 Ct) [range 27 to 948], respectively, over the P30-PCR (Figure 1). Contrarily to Wahad *et al*. [21], we did not observe B1-PCR positive and RE-PCR negative samples, and we confirm here that RE is the best target for toxoplasmosis investigation in pregnant women [16].

As the current microsatellite typing method is based on single copy targets [8], the use of a multicopy gene such as *B1* was expected to improve the number of typable samples. Using the *B1* gene, we increased the number of typable infecting *T. gondii* from 67% (49/73)—expected given the ability to amplify a single copy gene—to 86% (63/73). However, the *B1* gene’s sensitivity does not compete to that of RE. Of the 73 AFs available for genotyping studies, minisequencing analysis was not possible in 10/73 (13.7%) samples. These 10 non-genotypable AFs exhibited a RE-PCR Cq ranging from 32.4 to 36.3, *i.e*., a low quantity of *T. gondii* DNA. As a consequence, we expected 10-15% of non-typable samples to return positive only when using the RE-PCR assay. The 10 non-genotypable AFs were not associated with a given trimester (seroconversion in second trimester n = 3, third trimester n = 6, and unknown n = 1; p value = 0.26). These 10 non-typable AFs were all from France. This possible association of low amount of *T. gondii* DNA with French AFs might be due to comparatively early diagnoses due to the monthly mandatory screening of seroconversion during pregnacy instaured in France for several decades (see below).

**Figure 2:**
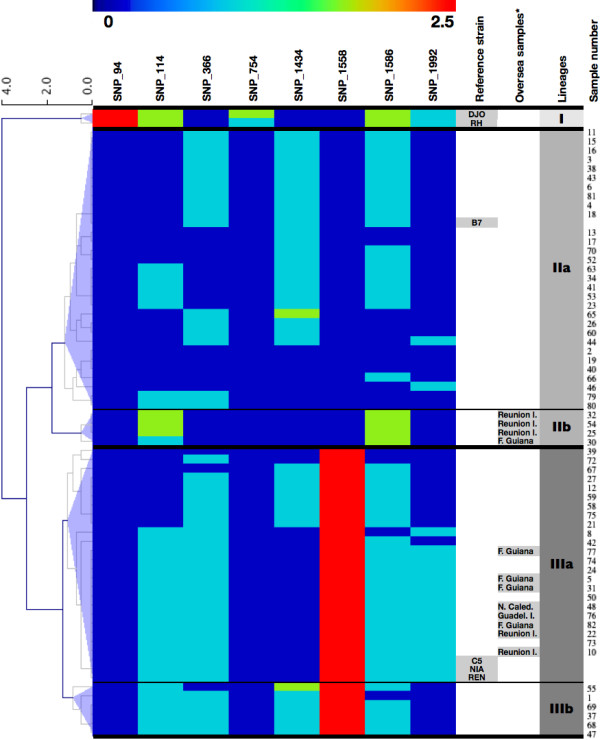
**Hierarchical clustering of the matrix generated for the eight tested SNPs using minisequencing analysis for 63 B1-PCR positive AFs and six reference strains.** Clusters corresponding to the well-known lineages I, II and III were delineated. Using the arbitrary threshold of 1.225, two additional clades could be separated in lineages II (IIa and IIb) and III (IIIa and IIIb). The names of the reference strains, the origins of the samples, the names of the clades, and the number of the samples are indicated on the right. Clustering was calculated based on Euclidian distance metrics and complete linkage. *Reunion I.: Reunion Island, F.guiana: French Guiana, N.Caled.: New Caledonia, Guadel.I.: Guadeloupe Island.

Genotyping based on B1 polymorphism was available for the 63 B1-PCR positive clinical samples and the six reference strains used, for which the DNA was extracted from pure culture. For each of the eight SNP markers, alleles were classified as wild-type, polymorphic, or mixture. Based on this classification in three different states, 25 genotypes were observed, with two genotypes observed only in the reference strains DJO and RH (Figure 2). Thus, although targeting a single locus, the *B1* gene’s rate of observed polymorphism was high, with 46% (25/69) of unique genotypes, confirming the marked genetic diversity of the parasite [3]. Other DNA polymorphisms were observed in *B1* and could be explored in order to increase discriminatory power if needed [10]. Based on a transition/transversion matrix analysis, hierarchical clustering distinguished the three major lineages (Types I, II, and III), as a reference strain of each lineage clustered in each of the three groups. Using an arbitrary threshold of 1.225, two clades were observed in lineages II (IIa, n = 30 and IIb, n = 4) and III (IIIa n = 23 and IIIb n = 6). The Clade IIa contained the reference strain B7, and the Clade IIIa the reference strains C5, NIA, and REN. No clinical sample clustered with the Type I reference strains (DJO and RH). Therefore, our system clearly distinguished the RH strain and atypical strains from Type II and Type III strains.

The repartition of the French and overseas samples was significantly different in Clades IIa, IIb, IIIa and IIIb (Figure 2), with an overrepresentation of overseas isolates (p < .0001) in Clade IIb (4/4, 100%) and Clade IIIa (9/22, 36,4%). Among the 56 samples for which data was available, the repartition of medical interruption and fetal death versus no abnormality significantly differed in the four clades, with an overrepresentation (p = 0.049) of medical interruption and fetal death in Clade IIb (2/4, 50%) and Clade IIIa (4/23, 17.4%). As already reported, the European infections were mainly due to Type II strains [7,8], whereas strains from French Carribean, French Guyana, and Reunion Island were confirmed to be more grave [4,6].

However, seriousness depends not only on the genotype, but also on numerous other factors, such as the time of infection [1,7]. In our study, we were able to confirm the association between medical interruption and fetal death and the first trimester of seroconversion (p = 0.0032). One might suspect that *T. gondii* infections during overseas pregnancies were diagnosed later, resulting in poorer outcomes. However, there was no association between the four clades and the date of seroconversion (p = 0.51). Similarly, seriousness was not associated (p = 0.33) with a geographical origin. Therefore, lower standards of care in overseas pregnancies are unlikely to bias the greater seriousness of some clades.

## Conclusions

Our study highlights the value of using the polymorphism of the repeated *B1* gene for directly genotyping *T. gondii* from clinical specimens without the use of any isolation or culture. Our typing system clearly individualized four clades, including the three main lineages of the parasite. Using the homogenous Type II genotype for background, we were able to individualize overseas genotypes with a higher virulence. However, we fully recognize that taxonomic studies with a single locus are limited, and therefore we did not attempt to further develop our markers in this context.

## Competing interests

The authors declare that they have no competing interests.

## Authors’ contributions

JMC designed the study, participated in molecular analysis and helped to draft the manuscript. AA performed the statistical analysis and helped to draft the manuscript. SM and VC carried out the molecular analysis. MD and JDP participated in conception of the study and in acquisition of data. SB coordinated the study, participated in analysis and interpretation of data, drafted the manuscript. All authors read and approved the final manuscript.

## Pre-publication history

The pre-publication history for this paper can be accessed here:

http://www.biomedcentral.com/1471-2334/13/552/prepub
